# Analyzing Spatial Patterns of Health Vulnerability to Drought in the Brazilian Semiarid Region

**DOI:** 10.3390/ijerph18126262

**Published:** 2021-06-09

**Authors:** Júlia Alves Menezes, Ana Paula Madureira, Rhavena Barbosa dos Santos, Isabela de Brito Duval, Pedro Regoto, Carina Margonari, Martha Macêdo de Lima Barata, Ulisses Confalonieri

**Affiliations:** 1 Transdisciplinary Study Group on Health and Environment René Rachou Institute–Oswaldo Cruz Foundation, Avenida Augusto de Lima, 1715, Barro Preto, 30190-009 Belo Horizonte, MG, Brazil; rhavena.santos@gmail.com (R.B.d.S.); isabelafbrito@gmail.com (I.d.B.D.); uconfalonieri@gmail.com (U.C.); 2 Department of Biosystems Engineering, The Federal University of São João del-Rei, Praça Dom Helvécio, 74, Fábricas, 36301-160 São João del-Rei, MG, Brazil; apmadureira@ufsj.edu.br; 3 Postgraduate Program of Meteorology, National Institute for Space Research, Rodovia Presidente Dutra Km 39, 12630-000 Cachoeira Paulista, SP, Brazil; pedro.regoto@yahoo.com.br; 4 Leishmaniasis Study Group René Rachou Institute–Oswaldo Cruz Foundation, Avenida Augusto de Lima, 1715, Barro Preto, 30190-009 Belo Horizonte, MG, Brazil; carina.souza@fiocruz.br; 5 Postgraduate Program of Public Health and Environment, National School of Public Health–Oswaldo Cruz Foundation, Rua Leopoldo Bulhões, 1480, Manguinhos, 21041-210 Rio de Janeiro, RJ, Brazil; baratamml@gmail.com

**Keywords:** vulnerability, drought, health, social determinants, rural population, Brazil

## Abstract

Health determinants might play an important role in shaping the impacts related to long-term disasters such as droughts. Understanding their distribution in populated dry regions may help to map vulnerabilities and set coping strategies for current and future threats to human health. The aim of the study was to identify the most vulnerable municipalities of the Brazilian semiarid region when it comes to the relationship between drought, health, and their determinants using a multidimensional index. From a place-based framework, epidemiological, socio-economic, rural, and health infrastructure data were obtained for 1135 municipalities in the Brazilian semiarid region. An exploratory factor analysis was used to reduce 32 variables to four independent factors and compute a Health Vulnerability Index. The health vulnerability was modulated by social determinants, rural characteristics, and access to water in this semiarid region. There was a clear distinction between municipalities with the highest human welfare and economic development and those municipalities with the worst living conditions and health status. Spatial patterns showed a cluster of the most vulnerable municipalities in the western, eastern, and northeastern portions of the semiarid region. The spatial visualization of the associated vulnerabilities supports decision making on health promotion policies that should focus on reducing social inequality. In addition, policymakers are presented with a simple tool to identify populations or areas with the worst socioeconomic and health conditions, which can facilitate the targeting of actions and resources on a more equitable basis. Further, the results contribute to the understanding of social determinants that may be related to medium- and long-term health outcomes in the region.

## 1. Introduction

The importance of socio-economic status and other underlying living conditions of the population has been considered relevant to public health policies and the reduction of health inequalities worldwide, especially after the Commission on Social Determinants of Health established by the World Health Organization in 2005 [1,2,3,4]. This approach recognizes the interaction between social, economic, cultural, ethnic, psychological, environmental, and behavioral factors that influence the occurrence of health problems and their risk factors in the population, creating health inequities among different strata. Recently, these determinants have been analyzed from the perspective of disaster risk reduction, since they influence and overlap the different elements that make up the risk, such as vulnerability, exposure, and adaptation, affecting the outcomes related to disasters and health of the population [5,6,7,8].

As are health outcomes and their determinants, the vulnerability to natural disasters is shaped by underlying risk factors such as poverty, urbanization, gender, and, more recently, climate change. Understanding that different types of hazards produce distinctive health burdens is then central to adequately design multi-sector measures to reduce disaster risks, although this might be a difficult task of implementing in extensive events such as droughts [6]. Although droughts are not the most common type of disaster, it was responsible for the highest number of deaths worldwide between 1900 and 2019 (about 30%) and has in a changing climate an additional risk factor [9]. In fact, critical changes in precipitation and temperature are expected for places already marked by this event, even if the target of warming up to 1.5 °C to 2 °C is reached [10].

Understanding how possible interactions between drought and health take place at the regional level is then essential to map risks and vulnerabilities, assisting in the agreement on adaptation and preparedness measures that contribute to reducing future risks from disaster and climate change to human health [11,12,13]. However, monitoring the outcomes of mid-to-long-term events such as drought makes evidence on the direct and indirect impacts scarce [6,8,13,14,15,16,17,18]. Most of them are indirect and long-lasting with multiple causal pathways, which hinders the establishment of a clear health-drought association due to, in part, the silent evolution of the event and its diffuse spatial distribution [14,16,19,20,21].

While scarce, epidemiological evidence shows the impacts of drought on human health ranging from an increase in infectious diseases to mental health deterioration [8,13,14,16,17,18,21,22,23,24,25]. A worldwide review from Stanke et al. (2013) [16] observed effects related to nutrition (e.g., mortality and malnutrition), water-borne diseases (e.g., cholera, algae bloom), and vector-borne diseases (e.g., malaria, dengue). Local studies have shown possible effects related to nutritional deficiencies, mental health, water and air quality, compromised quality and access to health services, and slower gains in population health, perpetrating long-lasting consequences of drought to human well-being [8,14]. Water and food security, social capital, and social determinants have also been related to health vulnerability to droughts in different regions, including semiarid places [14,15,21,26,27,28]. However, especially in Brazil, these studies are often limited to the biophysical and epidemiological impacts of disasters, failing to produce a bigger picture on the theme [8,13,14,15,17,29,30,31].

In this sense, the use of indices focused on understanding health risks in all its dimensions may be valid for a better understanding of the distribution of local health outcomes and can add valuable information to identify health vulnerabilities useful for disaster risk reduction [32]. Indices related to social vulnerability, human health, climate change, and infectious diseases are a common practice in disaster risk and public health approaches, adding to the comprehension of important underlying health risks, highlighting inequalities in the epidemiological profiles of populational groups, and prioritizing public health resources for slow onset disasters in specific areas [14,27,32,33,34,35,36,37,38,39,40,41,42].

The present study can add a multidimensional perspective to this context, highlighting complex interactions basing the drought–health relationship in Brazil, as it brings together the perspectives of environmental disasters, social determinants, and possible health effects in an index useful for vulnerability analyses. The findings might provide evidence of the underlying health–drought connections in the Brazilian Semiarid municipalities, a region considered the most inhabited semiarid area on the planet (more than 22 million people). Based on a multivariate analysis, this study proposes (i) to identify how some important determinants of health related to drought are grouped and distributed; (ii) to identify vulnerable populations by creating a relative vulnerability index that produce a spatial view of the health–drought patterns in the region.

## 2. Materials and Methods

### 2.1. Study Area

**Figure 1:**
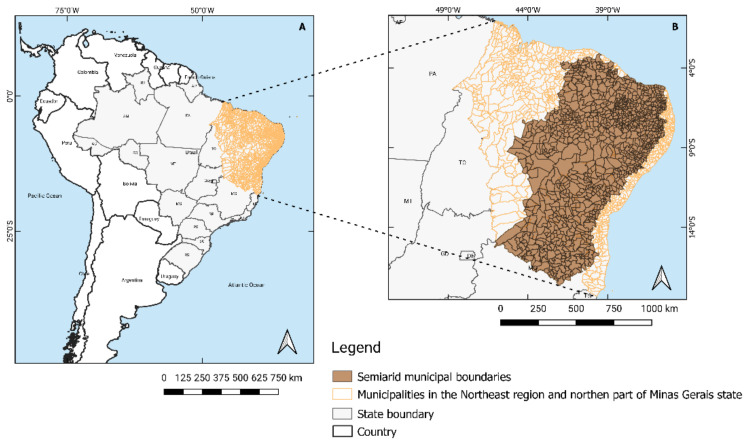
Limits of the study region—Brazilian Northeast region and part of Minas Gerais state (**A**), and the Semiarid municipalities (**B**). The 1135 municipalities studied are located mainly in the Northeast region of the country, but also occupy part of the northern portion of the state of Minas Gerais, in the Southeast region (85 municipalities). Adapted from: [31].

The Brazilian Semiarid region is situated mainly in the Northeastern part of the country, being delimited based on the following dominant semiarid climatic conditions: (i) average annual rainfall below 800 mm; (ii) aridity index of up to 0.5 (water balance between precipitations and potential evapotranspiration); and (iii) drought risk greater than 60%. It has 22,598,318 inhabitants (about 12% of the Brazilian contingent), underperforming the other regions in key indicators such as illiteracy, infant mortality, and poverty [14]. In addition to this social context is the scarcity of natural resources and the poor agricultural and livestock production, negatively affecting the living conditions of communities, which have in subsistence farming one of their main economic activities [13,30,31]. The rainfall has a strong space–time variability (concentrated in 3–4 months) and low total annual volumes (average accumulated precipitation less than 600mm year-1, which are more reduced in the interior parts of the region) [43,44,45]. Droughts are a chronic phenomenon registered since at least the 16th century, the most recent lasted from 2010 to 2016 [46]. Large-scale phenomena like El Niño and La Niña are often associated with exceptionally dry or wet episodes in the region [46]. The present study was based on the 2005 delimitation, which includes 1135 municipalities in nine states of the federation—most of which are located in the Northeast region of the country, while a few occupy the northern part of the state of Minas Gerais (Figure 1).

### 2.2. Conceptual Framework

In public health, vulnerability represents a multidimensional construct comprising several biophysical, sociocultural, political, institutional, and economic factors that converge at the community and individual levels to influence health outcomes. It also represents a dynamic process that acts to modulate the capacity of populations or systems to cope with adverse impacts of extreme events, being influenced by underlying factors known as determinants of health [1,13,31,47,48]. In the literature of disasters, these health determinants are considered key in understanding population level outcomes following disasters, and are known as determinants of vulnerability [48,49]. These key conditions help in understanding existing vulnerability prior to disasters, which can also be exacerbated after a disaster, fulfilling priority one in the Sendai Framework of addressing disaster risk from location-based information [7,48,49].

**Figure 2:**
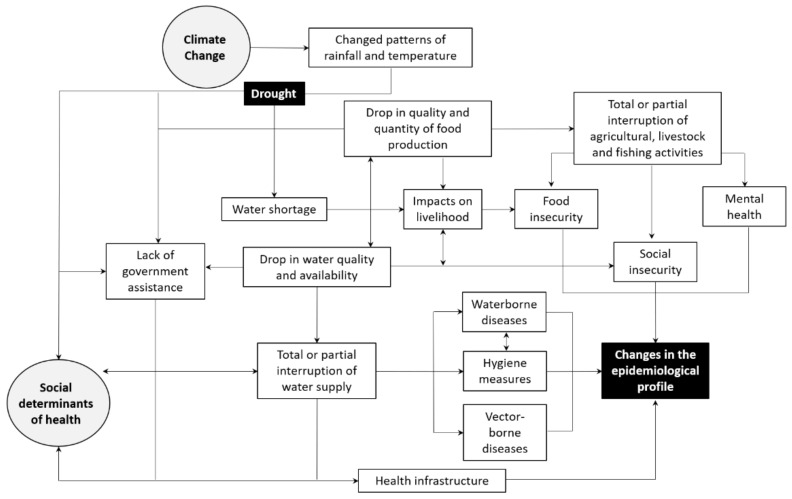
Conceptual framework on the links between drought, health, and the environmental and social determinants. Adapted from: [8,16,27].

Considering these key concepts, an explanatory model was developed for the Brazilian Semiarid region. Figure 2 represents the possible and complex interactions that are established between sociodemographic, environmental, and health aspects in the context of droughts. It is noticed that the health effects occur slowly and mostly indirectly so that the vulnerability of the population is shaped by factors such as location in the geographical space, the subsistence economy, and the lack of government investment in mitigation and assistance measures that may impact health [27,50]. In general, changes in rainfall and temperature affect the quantity and the quality of the water available for consumption, producing a cascading scenario of food and social insecurity, damage to health infrastructure or human resources, and other health issues that can modulate the epidemiological profile of the population. The direct and indirect impacts arising from drought influence other determinants of health (e.g., socio-economic vulnerability), as well as being influenced by them, resulting in changes in the population’s health status.

### 2.3. Vulnerability Data

The 32 indicators were compiled, and their sources are shown in Table 1. A municipal scale was chosen, comprising 1135 study units in the Brazilian Semiarid region. The initial categorization of variables was based on the structure proposed by Ebi & Bowen (2016) [28] to explore the main health vulnerabilities in the context of extreme weather events, namely, socio-environmental, socio-economic, and health status/health systems. The criteria for inclusion of variables were: (i) to demonstrate a literature basis of the relationship between health, its determinants, and vulnerability to drought, and (ii) be available on systematic and freely accessible platforms (i.e., public, governmental, or private).

### 2.4. Multivariate Analysis

An exploratory factor analysis was performed in order to obtain groups of indicators more correlated with each other. This type of multivariate procedure is used to obtain latent variables (factors) that would not be observed directly in the data set, allowing the creation of scales or indexes through its output scores [51,52].

The entire database was prepared to replace missing or no variation values as required by statistical procedure. In the case of missing values, the mean value of the variable was used, which did not change its distribution. All information was normalized by the minimum–maximum method to present the same scale—the minimum value is transformed into a 0, the maximum value is transformed into a 1. Model adequacy measures comprised the subject–item ratio, sample size, Bartlett sphericity, and Kaiser–Mayer–Olkin tests [53,54,55,56,57]. The extraction method used was the Iterated Principal Factor (IPF), indicated when the multivariate normality of the variables is not met [55]. Orthogonal (varimax) and oblique (oblimin) methods were chosen for rotation. For the factors retention, the following rules were followed: Kaiser criterion, scree plot, and parallel analysis as quantitative methods, in addition to the gradual elimination of factors (stepwise) and the interpretability of the results [51,52,58]. The data analysis for this paper was generated using SAS^®^ Studio Software, Version 3.8 (SAS Institute Inc., Cary, NC, USA) [59].

### 2.5. Indices and Maps

The standardized scoring coefficients created by the SAS^®^ during the factor analysis were used as weights to generate a Health Vulnerability Index (HVI) in drought situations. The normalized variables were multiplied with the assigned weights to construct separate indices for each common factor, using the regression method with the following formula:

(1) $$I_{j}=\sum_{i=1}^{k}b_{i}\left[\frac{a_{ji}-x_{i}}{s_{i}}\right]$$

where, *I* is the index value of the factor, *b* is the standardized scoring coefficient (weights), *a* is the indicator value, *x* is the mean indicator value, *s* is the standard deviation, *i* is the indicator, and *j* is the specific municipality. Thus, an underlying index of vulnerability, per municipality, was created considering only the indicators that comprised each factor. All indices were normalized using the minimum–maximum method.

A simple additive model of the four factors was used to generate the HVI, with no weights assigned, allowing each factor to contribute equally to the overall vulnerability score [38]. It being an exploratory analysis, the approach of equal weights was chosen because there was no prior assumption about the importance of each factor for the overall sum. A cardinality adjustment was performed to demonstrate the influence of each factor on the final vulnerability [38,52,60,61]. For this purpose, the relationship of the indicators with the vulnerability and the value of their factorial loads was analyzed, being adopted mainly, but not exclusively, the limits of 0.7 or 0.5.

The QGis software, version 3.10 “A Coruña”, was used to spatialize the indices in choropleth maps and allow a visualization of their regional distribution. The maps varied between 0 and 1, indicating a comparative scale from the lowest to the highest vulnerability, respectively, for the municipalities of the Semiarid.

## 3. Results

The model’s adequacy measures were satisfactory. The subject–item ratio was 32:1, and Bartlett’s sphericity test (*p* ≤ 0.001) and Kaiser–Mayer–Olkin test (KMO = 0.82) presented values suitable for analysis. The Cronbach’s alpha value was 0.7 (most of the variables demonstrated robust internal consistency with Cronbach’s alpha ≥ 0.6 for each). The four-factor solution showed consistent results between the initial sample (*n* = 1135) and the random samples (*n*1 = 567 and *n*2 = 568), demonstrating the stability and reliability of the initial solution.

Four common factors were retained, a result converged in all quantitative methods (Kaiser factor, parallel analysis, and scree plot). Together, these factors explained 84.4% of the variance observed for the 32 indicators included. An oblimin rotation verified low correlations between the factors [62]. Hence, the varimax rotation model (i.e., assumes independent factors) proved to be more appropriate since one of the aims of this work was to generate indices for each Semiarid municipality. The results for factor analysis are shown in Table 2.

Health and its social determinants represented the first common factor with the highest proportion of explained variance (51.8%). Some living and socio-economic conditions of the municipalities of the Semiarid region that, in the public health context, are known to influence the occurrence of health problems and its risk factors were highlighted. These conditions are mainly related, but not limited, to income, education, and quality of life, features with loads greater than 0.5. While the highest per capita income and higher education were positively related to the factor, other important indicators were negatively related: occupation in agriculture, low income, and low education. Therefore, the cardinality of the factor has been reversed to reflect the greater vulnerabilities in places with poor socio-economic and health conditions.

Rural Economy and access to water represented those municipalities with higher prevalence of rural activities, explaining 13.2% of data variance. The municipalities with the highest proportion of rural households with water related technologies were those in which the availability of the water supply system is lower, increasing overall vulnerability. Although the existence of water technologies represents less susceptibility to drought, a data analysis on this factor reveals this indicator positively correlating to the rural population and the drought index, but negatively relating to irrigated farming and piped water, pointing out the most critical locations from the point of view of water scarcity. Thus, this factor was considered to increase vulnerability.

Factor 3 represented health problems and infrastructure with 10.4% of the explained variance. It indicates those municipalities where the burden of hospitalizations for health conditions that may be related to droughts is greater, as well as demonstrates the effectiveness of the care provided to the population (i.e., beds and primary health care). Hospitalizations for conditions related to drought phenomena in the scientific literature—asthma, malnutrition, and diarrhea (the first with the highest load)—and admissions sensitive to primary care, were grouped together, all with positive loadings. Another less relevant information was the number of beds per 1000 inhabitants. All the characteristics observed in this factor increase vulnerability in drought contexts, except the number of beds, hence the cardinality was positive.

Finally, the fourth factor was related to the characteristics of rural establishments, named as rural structure and social capital (explained variance of 9.2%). The main attributes were the prevalence of family farming with a positive load and households headed by illiterate women with a negative load. The interpretation of this factor indicates that the places where there is a greater participation of family farming are those where there is some social organization, properties with a watercourse and fewer women as breadwinners. Although it seems contradictory to hold a positive cardinality for this factor, family farming is very important in rural semiarid regions, as it represents a low productivity activity focused on subsistence and is very susceptible to droughts, which is why this factor was considered to increase vulnerability.

**Figure 3:**
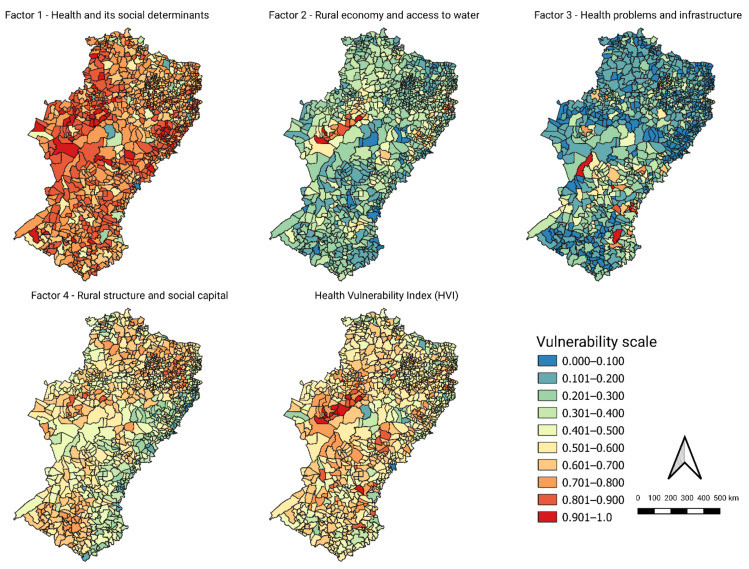
Spatial distribution of the Health Vulnerability Index (HVI) and its factors for municipalities of the Brazilian Semiarid region ranging between 0 and 1 (0 represents lesser vulnerability and 1 greater vulnerability).

The spatial distribution of the factors, as well as the HVI are shown in Figure 3. There is a large regional difference in the distribution of vulnerability between the four factors. Health and its social determinants were found to be underdeveloped throughout the Semiarid region, with municipal clusters presenting values greater than 0.8 in the extreme west towards the north, and at the eastern border towards the south. The municipalities with lower values in the index were dispersed. For rural economy and access to water (Factor 2) there is a general reduction of vulnerability, where most of locations ranged from 0.2 to 0.6. Clusters of municipalities with low values prevailed in the southern and northern regions, while municipalities with less developed rural economies concentrated in the western and northeastern parts. A similar situation was observed for health problems and infrastructure, in which vulnerability was punctual, especially in the center–south portion. Most municipalities were placed in categories of lesser vulnerability, with groups less vulnerable to health issues in the southern and northern parts of the Semiarid region. Factor 4, in which the conditions of rural establishments were highlighted, showed a tendency to increased vulnerability from the eastern border, which assembled vulnerabilities below 0.3, and a dispersion of the highest scores towards the northern, central–western, and southern areas. It is worth mentioning that in this factor, the eastern belt, of lesser vulnerability, represents those municipalities in which there is a greater concentration of illiterate female breadwinners, with little rural social articulation and poorly developed family farming, that is, they are more urbanized or present commercial agriculture, at the expense of subsistence farming.

The HVI represents the results of the additive model and reflects some of the patterns found in the factors individually. There was a tendency for high scores to be concentrated in the central–western and other clusters in the northeastern and southern portions. However, it is perceived a modulation of the different aspects of vulnerability in the HVI through the “compensation” between the factors, which seems to have leveled the extremes. Thus, although for health and its social determinants (Factor 1), the highest vulnerability scores prevailed across the Semiarid region, with special attention to the extremes of the border (eastern–western), the other factors modulated the final vulnerability by presenting lower vulnerability scores for many of these critical regions. All the index scores are available at a data repository [63].

## 4. Discussion

### 4.1. Rural and Social Characteristics Influencing Health Vulnerabilities

The literature on vulnerability, health and droughts in Brazil has been growing in recent years, but the discussion on health determinants of disasters and drought vulnerabilities is still scarce [8,13,14,15,29,31,64]. In this sense, the present study proposed an index of health vulnerability in drought situations (HVI) based on social, economic, epidemiological, and environmental indicators that, assembled in factors, elucidated some health determinants, based mainly on publicly available information for all the Semiarid municipalities.

In general, the configuration of the factors of this study showed rural characteristics in a remarkable aggregation with worst living conditions. In Factor 1, the population employed in agriculture was opposed to higher levels of education and average income per capita, while aligning to income below the poverty line and illiteracy. Factor 2 showed conditions such as drought, rural population, and water technologies in a opposite direction of access to irrigated agriculture and access to piped water. This might explain why the municipalities with the highest values in this index presented a higher proportion of cisterns and other forms of water storage. In Factor 4, family farming showed greater weight in the definition of the factor, a condition considered extremely susceptible to environmental hazards such as drought, while antagonistic correlating to women heads of households, a predominant situation in urban areas of the Brazilian Northeast [31]. All these findings point to the possibility of rural subsistence conditions acting as a predictor of increased health vulnerability and poor quality of life in the region. Furthermore, it allows differentiating the municipalities between those with predominant urban characteristics, where rural economies are better developed (commercial agriculture), and those essentially rural, with subsistence farming as major activity.

Similar findings were reported by several authors in the region [31,65,66,67]. Hummell, Cutter, & Emrich (2016) [39], when replicating the Social Vulnerability Index (SoVI^®^) in Brazil, observed that the population employed in agricultural activities and livelihood was linked to greater social vulnerability and less developed areas. In the same sense, the National Institute of the Semiarid (INSA), while monitoring the desertification process in the region from a model considering institutional, economic, and social drivers, pointed out as prone to desertification, the same areas indicated as the most vulnerable in Factors 1 and 4, therefore less liable to commercial agriculture [68]. These locations were the ones where social determinants of health presented impoverished and the family farming prevailed.

Although the Northeast, where most of the Semiarid is located, has made improvement in its socio-economic conditions, health sector activities, and supply services since the 2000s, investments and the expansion of economic activity maintained the historical trend of concentration in state capitals and in traditional regional hubs [69]. This has produced inequalities in access to agricultural technology and resources, concentrating these assets in environmentally and economically prosperous areas, while those with predominance of subsistence agriculture face greater difficulties imposed by drought. Thereby, these locations end up demanding more social and political articulation to ensure better living conditions and commercial competition, which might explain why the social organization indicator is positively correlated to family farming in Factor 4. Additionally, the indicator of irrigated agriculture, by showing to be inversely correlated to the rural population and the incidence of drought in Factor 2, shows that this type of technology is not available or is not viable for the places most vulnerable to drought in the Semiarid. Indeed, family farming is more common in smaller tracts of land in the region and is based primarily on rainfed systems, an activity very vulnerable to water scarcity and characterized by low levels of productivity, which makes small farmers highly vulnerable to drought impacts [70]. However, irrigation proved to be effective in managing risks in the context of drought in Northeast, where families in possession of this asset were less likely to experience food insecurity than families without irrigation [71].

These findings align with other studies showing that, both globally and in Brazil, the rural population is often more exposed to drought hazards than urban populations [26,72]. Rural households are at greater risk of chronic food insecurity than families in urban areas, for example [73,74]. Overall, rural societies earn lower incomes and may be more dependent on natural resources and the local economy, being particularly susceptible to climate hazards [26,38,39,71]. In drought situations, the cycle of reducing subsistence, decreasing income, and increasing prices of agricultural products, in a scenario of low rural technologies as observed in the Semiarid, is seen as a driver of food insecurity in this populational group [30].

The deprivation of access to drinking water is another problem added to this resource scarcity scenario, where the countryside is usually the most affected area. This condition was demonstrated in Factor 2, in which access to piped water has been shown to be inversely related to the rural population and to the greater incidence of droughts. Several government and civil society programs have sought to minimize the effects of drought by making water tank truck operations and water storage programs a commonplace. Programs such as “cisterns”, “one million cisterns”, and “one land two waters” have been implemented to provide access to water for human consumption and food production, engaging simple and low-cost social technologies [29,75]. However, such efforts have not been enough to tackle the reality experienced by agricultural families, since the poor water quality provided by tank trucks and cisterns, is combined with other problems such as lack of sanitation, with significant health repercussions.

Worldwide, health indicators tend to vary according to the social gradient, being less favorable in groups of lower socio-economic levels, whether measured by income, education, occupation, or social class [3,48,76,77,78,79]. These indicators are often related in an “ecological level”, in which spatial clusters show areas with a high income level offering good coverage of sanitation services, health facilities and education with high populational density [80,81,82]. This relationship was observed in Factor 1 of the present study. A polarization was observed between locations with more concentration of wealth, population, and human well-being (e.g., higher income, education, health coverage, and sanitation), and places with limited resources, where residents with lower quality of life and health are found (e.g., lower income, illiteracy, higher agriculture labor force and infant mortality).

This distinction highlights persistent intra-regional discrepancies in the Semiarid, albeit this region is considered quite homogeneous in its socio-economic and health levels [3,13]. This can be explained by the region’s development profile, influenced by the migration of labor force to large regional hubs. In these places, the population density was not accompanied by the expansion of public services infrastructure, fostering urban agglomerations with poor living standards than the national average, while the countryside remained lacking investments [83]. However, underneath this apparent homogeneity, local dissimilarities still emerge as identified by other authors for economic, land use, and health indicators [13,14,84,85,86,87]. Factor 1 ends up locally reflecting this bigger picture where poor human well-being and social/health inequalities are observed for the whole Semiarid region, strongly distancing it from the patterns observed for other Brazilian regions, while highlighting its local differences.

Regarding health issues, the municipalities with the highest level of health care development were those with the best socio-economic performance (observed in the Factor 1 arrangement). Health professionals per capita and coverage of private health plans showed a positive correlation with indicators such as average income, higher education, access to sanitation, and lower infant mortality rate. On the other hand, some of these characteristics have been positively correlated to morbidity indicators such as dengue, hepatitis, mental disorders, and skin infections, even though featuring small loads. This fact seems to demonstrate that even the most prosperous Semiarid areas, in which the social determinants of health have shown better scores, still lack investment in health care and health promotion actions. Yet, recent socioenvironmental changes such as urbanization, population growth, poverty, and climate change pose an additional risk to the proper management of impacts related to extreme events as droughts, since they could modify the emergence and transmission of infectious diseases and other health problems [88,89].

Although Factor 3 has been shown to be quite homogeneous regarding the distribution of health problems and infrastructure across the municipalities, poor values were observed in the center–southern portion. In these places, the health outcomes that may be related to drought represented a great burden to the health system, with higher admission rates. However, the availability of beds with positive loads suggests that these municipalities are also able to offer more complex health services. On the other hand, high loads were obtained for hospitalizations sensitive to primary care, showing that many health problems are transferred to more complex levels when they should be addressed at the entrance level of the system. It highlights the current deficiencies in primary care and the likely overloading of secondary care, contributing to a lower resolution of local health services. These municipalities may also have a reduced capacity to respond and cope with extreme weather events due to the great burden on the health system, since other common health outcomes not directly related to droughts, such as hypertension and diabetes, are managed mainly at primary care levels.

The Unified Health System (SUS) represents the only assistance structure for a large part of the Semiarid population, mainly for rural, which still face recurring difficulties in accessing health care services [3,15,90,91,92]. The infrastructure and health care networks, at its different levels of complexity, shape to a certain extent the assistance available to the public along to the allocation of resources in Brazil. Primary care is present in almost all municipalities, while secondary and tertiary levels are available in regional or large urban centers. Such an arrangement fosters local inequalities in the allocation of financial and human resources, as well as in the availability of medical and hospital services, equipment, and instruments. This generates a shortage of physicians and other specialties in small rural areas and in primary care levels, while concentrating specialists in the private sector of large urban hubs [69,91,92]. As small municipalities are predominant in the Semiarid, health services and access to them are restricted to more prosperous regional hubs with better conditions for attracting and retaining health professionals, which also provide better infrastructure, better collective working conditions, higher income level, and higher quality of life [29,90,93]. It is possible that such aspects contributed to the spatial homogeneity of Factor 3.

### 4.2. Social Determinants at the Borders, Rural Aspects in the Inland Regions

Spatial analysis proves to be an important instrument in assessing the impact of social processes and structures in determining disaster vulnerabilities, highlighting the municipalities in which health determinants—environmental, economic, and social—must be better analyzed. Starting from an overview of the HVI, the highest values were shown to be continuously grouped on the eastern and western borders, where the municipalities with the least social and economic development are located. At the same time, most municipalities remained in intermediate categories of vulnerability in HVI—between 0.3 and 0.7—demonstrating a homogeneity of health conditions and its determinants as observed by other authors [13,39]. This arrangement causes the municipalities of the Semiarid region as a whole to present a widespread fragility of the health system, somewhat demonstrable by the worst health conditions observed amid the resident population, which are not shared by other non-semiarid municipalities in Brazil [14]. This pattern is also apparent in other indices or studies adapted to the Brazilian reality such as the Municipal Human Development Index, the SoVI^®^ and others [13,14,39,94,95]. Usually, intermediate levels of development and vulnerability prevail in the Semiarid, with a general worse performance when compared to the rest of the country.

The factors that presented the most dispersed vulnerabilities in the semiarid territory were the health and social determinants and the rural structure, with the first presenting a greater homogeneity in the spatial distribution of the highest vulnerabilities. This lack of clear differences was expected due to the unfavorable socioeconomic conditions of the Semiarid as a whole, which comprised central characteristics of the social determinants of health grouped precisely in Factor 1. The opposite could be observed for Factors 2 and 3, in which the spatial pattern of lesser vulnerabilities prevailed in the territory.

A general pattern of poor values grouped on the frontiers for health and its social determinants index (Factor 1), while the other factors modulated the final vulnerability (HVI) by presenting lower vulnerability scores was observed. An overlap between the areas of greatest vulnerability in each factor was evident, except for Factor 3. Consistently, a group of municipalities in the western portion of the Semiarid was amongst the largest categories of vulnerability of health and its determinants, rural economy, and rural structure, stressing the need for investments in adapting to droughts regarding the socio-economic and water access levels. These localities comprised small towns, with a high dependence on external revenues (i.e., those coming from other sources such as the state and union), with low income from work, high rates of infant mortality and with medium to high levels of previous droughts records [13,96].

In Factor 2, where the most vulnerable categories represented the places where there is a greater rural population, with a reduction in agricultural participation in GDP, less irrigation and less access to piped water, there is an intersection with the greatest vulnerabilities of the health and its social determinants. Similarly, the spatialization of Factor 4 reveals the highest values associated with the municipalities with the greatest percentage of family farming, although with some type of social organization (western part), locations where Factor 1 also presented high scores. Thus, western, eastern, and northeastern parts of the region represent places where the health of populations, in its various aspects, may be more affected in the context of drought, lacking social investments, with poor quality of life, low income, and precarious access to water that increase their vulnerability to drought.

Similar conclusions were reported by Vieira et al. (2020) [31] regarding vulnerability of the drylands. The authors showed that physical characteristics of dry regions do not necessarily imply high social vulnerability, but rather a historical political environment that defines the social construction of risk associated with droughts in Brazil. The fact that the drought indicator was not among the largest loads in Factor 2 points in the same direction—although the whole Semiarid experiences the impacts of droughts at diverse intensities, are the aspects of infrastructure, services, employment, income, and social conditions more important in shaping the HVI. These conditions are probably a consequence of both poor management and reduced political will in facing the recurrent droughts of the region, rather than a consequence of the climate hazard itself. However, the current scenario can be greatly exacerbated by the ongoing climate change, given that changes in annual-mean air temperature are projected higher for the Brazilian Northeast than globally, demonstrating that local impacts can be much stronger [97]. Sectors already weakened, such as food and water security, as well as small-scale agriculture, can be strongly impacted by warming above 4 °C due to increased temperatures and reduced precipitation, increasing the vulnerability of smallholder livelihoods in municipalities supported by subsistence agriculture [97].

Interestingly, the HVI spatial pattern was remarkably similar to the risk index constructed by Sena et al. (2017) [13] for the Semiarid considering drought situations. The similarities also encompass the distribution of their vulnerability and access to piped water indices, which presented extensive overlap with the health and social determinants index of the present study. This corroborates the factors pointed out here as fundamental to shape health vulnerabilities to droughts in the Brazilian Semiarid and to help in reducing existing hazards, while creating resilience to future ones. Key factors that must be tackled at the populational level to lessen drought impacts before and after its occurrence were highlighted.

However, some limitations of the study should be addressed. They refer mainly to (i) the weighting scheme used to calculate the HVI and (ii) the use of secondary data and its systematization. It is acknowledged that different methods lead to significantly different results, directly affecting the value of the index and shifting considerable the ranking of the municipalities under study [98,99,100]. This is important when dealing with vulnerability indices and its implication to decision making, as different spatial vulnerability pattern related to the chosen methodology may be used by different actors (e.g., authorities, planners, and emergency services) [98]. However, even though there is a lot of discussion about the robustness of the different weighting schemes available, the HVI methodology can be a good starting point in supporting the Semiarid municipalities to ascertain the similarities and differences in their relative levels of health vulnerability. Regarding the data limitation, SUS official data refer mainly to the public sphere, which, although comprising most of the information, does not express the totality of the health indicators in the region. Another aspect is the contemporaneity of socio-economic data, which, despite being systematically updated, may have long-term intervals between your publications (e.g., census editions). It is possible that the use of more recent indicators provides a better representation of health vulnerability in the context of drought. However, this update is feasible from the release of new data, adding to the HVI and its factors an ability to demonstrate the evolution of health vulnerabilities overtime.

## 5. Conclusions

This study developed a health vulnerability index for the Brazilian Semiarid region from a factor analysis that showed the connections between different aspects influencing the health vulnerabilities in the context of drought. Investment in improving education, employment and income, healthcare facilities, family rural production, and access to water proves essential to ensure the quality of life and health of the population. Moreover, the conformation of the factors made it possible to distinguish the municipalities between those with subsistence characteristics and those with developed commercial agriculture, with marked differences in human well-being. The simple approach of the method helps understand the dynamics of the relationship between health and its determinants, as well as contribute to the spatial visualization of the associated vulnerabilities. The results might support decision making on drought risk reduction as the identified determinants are modifiable underlying conditions, which are linked to medium to long-term health outcomes arising from disasters.

It is believed that the built indices can serve as a guiding tool for decision making at regional levels, helping to reduce risk and increase local resilience of the public health sector. Monitoring the changes that are anticipated in the indices proposed here, from its systematic update, can secure the adequate management of health outcomes more related to drought in the Brazilian Semiarid. Future directions point to need of continued efforts in examining the health–drought nexus in the region, bringing together stakeholders and policy-makers’ perspectives to build a local fit approach to tackle health vulnerabilities in the context of the ongoing climate change. Further, an extension of the study is possible as the region presents now a more recently political delimitation, which comprises 1262 municipalities distributed throughout 10 Brazilian states. This might help in a more rational and direct application of the HVI in guiding regional policy practices in the region.

Appropriate policies to ensure the improvement of health determinants and drought vulnerabilities are needed, mainly those related to rural areas and human well-being. Furthermore, the capillarity of the health sector shows itself crucial in various governmental instances, as it enriches the debate about the most visible or long-term impacts associated with droughts. It also assists other sectors whose actions directly affect the quality of life of the population, such as agriculture, water management, and social protection, enabling people to increase control over, and to improve, their health.

## Figures and Tables

**Table 1:** Description of the variables and indices.

Dimension	Indicator	Variable	Source
Socio-economic	Income below the poverty line	% of households with per capita nominal monthly income (BRL) of up to 1/2 minimum wage in 2010	Demographic census–IBGE
	Per capita income	Value in BRL of average household income per capita in 2010	Demographic census–IBGE
	Ratio between rural and urban population	Resident population whose household situation was rural in 2010	
		Resident population whose household situation was urban in 2010	
	Population with complete primary education or more	% population aged 15 years or older with a completed 2nd elementary school or more in 2010	
	Illiterate population	% population aged 15 years and older with no education in 2010	
	Survival probability	Likelihood of a newborn child living up to 40 years if the level and pattern of mortality by age of the 2010 Census remain constant throughout life	Atlas of Human Development in Brazil
	Illiterate female heads of household	% of households in which the woman was responsible and illiterate in 2010	Demographic census–IBGE
	Dependency ratio	% of people living in households with a dependency ratio > 75% in 2010	Atlas of Human Development in Brazil
	Unemployment rate	Unemployment rate of people aged 16 years and older in 2010	Demographic census–IBGE
	Population employed in agriculture	% of the employed population in the agricultural sector aged 18 years or older in 2010	Atlas of Human Development in Brazil
	Municipal population engaged in family farming	% of establishments presenting a declaration of suitability to PRONAF (National Program for Strengthening Family Agriculture) in 2017	Agricultural Census–IBGE
	Rural establishments where the producer is an association member	% of establishments in which the producer is associated with a cooperative or class entity in 2017	Agricultural Census–IBGE
	Rural establishments with irrigated agriculture	% of establishments with irrigated agriculture in 2017	Agricultural Census–IBGE
	Rural establishments with access to water	% of establishments with rivers/streams protected by riparian forest in 2017	Agricultural Census–IBGE
	Rural population with access to water technology	Number of rural households served by water access technologies (i.e., consumer cisterns, storage tanks) in 2019	National Semiarid Institute–INSA
Socio-environmental	Demographic density	Resident population in 2017	IBGE
		Municipal area in km^2^	National Semiarid Institute–INSA
	Drought index	SPI-12 frequency and duration. Methodology adapted from [48]. Standardized Precipitation Index (SPI) is the most commonly used indicator worldwide for detecting and characterizing meteorological droughts, based on a comparison of observed total precipitation amounts for an accumulation period of interest (e.g., 1, 3, 12, 48 months)	CHIRPS
		Number of drought events recorded between 2003 and 2015	National Water Agency—ANA
	Change in agricultural participation in gross domestic product (GDP)	Gross change in income obtained through work in the rural area between 1999 and 2012	National Semiarid Institute—INSA
	Population with access to sanitation	% of households with general sewerage or septic tank in 2010	Demographic census—IBGE
	Population with access to piped water	% of households with public water supply in 2010	
Health conditions and systems	Dengue index	Incidence rate, temporal trend, and proportion of cases between 2001 and 2015. Adapted from [26,31]	DATASUS
	Hepatitis A index		
	Asthma admissions rate	Hospital admission rate, temporal trend, and proportion of cases between 2001 and 2015. Adapted from [26,31]	
	Malnutrition admissions rate		
	Skin infections admissions		
	Mental disorders admissions		
	Diarrhea admissions		
	Admissions sensitive to primary care	% of hospitalizations for conditions sensitive to primary care in 2015 (a set of health problems for which the effective action of primary care would decrease the risk of hospitalizations)	
	Infant mortality up to 5 years	Probability of dying between birth and the exact age of 5, per 1000 children born alive in 2010	Atlas of Human Development in Brazil
	Number of beds/inhabitants	Total outpatient, emergency, intensive care, and hospitalization beds per 1000 inhabitants in 2015	DATASUS
	Health professionals per inhabitant	Number of registered health professionals in the public and private sectors per 1000 inhabitants in 2015	
	% population covered by health insurance	Number of health plan beneficiaries that contain hospital and/or outpatient segmentation, and may also contain dental assistance in 2015	National Supplementary Health Agency
		Estimated population in 2015	IBGE

**Table 2:** Factor names, indicators, percentage of explained variance, factor loads, and cardinality.

Factor Name	Indicator	Factor Loading	Explained Variance (%)	Cardinality
Health and its social determinants	Average household per capita income	0.803	51.8	+
	% population with complete primary education or more	0.705		
	% population covered by health plans	0.488		
	Survival probability	0.469		
	Demographic density	0.448		
	% of households with access to sanitation	0.427		
	Health professionals per 1000 inhabitants	0.421		
	Dengue index	0.393		
	Skin infections admissions	0.337		
	Hepatitis index	0.245		
	Mental disorders admissions	0.203		
	Infant mortality	−0.438		
	Dependency ratio	−0.459		
	% of the population employed in agriculture	−0.666		
	% households with monthly income per capita up to ½ salary	−0.685		
	% population illiterate	−0.693		
Rural economy and access to water	% rural households with water related technologies	0.639	13.2	+
	Rural urban ratio	0.456		
	Drought index	0.397		
	Unemployment rate	−0.315		
	% rural establishments with irrigation	−0.319		
	% change in agricultural participation in GDP	−0.354		
	% households with access to piped water	−0.707		
Health problems and infrastructure	Asthma admissions	0.783	10.1	+
	Undernutrition admissions	0.569		
	Diarrhea admissions	0.520		
	Admissions sensitive to primary care	0.520		
	Beds per 1000 inhabitants	0.387		
Rural structure and social capital	% family farming establishments	0.615	9.2	+
	% rural establishments associated with a cooperative or class entity	0.418		
	% establishments with water resources	0.312		
	% of households with female heads of household illiterate	−0.540		

## Data Availability

The data presented in this study are openly available in Mendeley repository at [http://dx.doi.org/10.17632/vfvrr97zb4.1].
